# Extending the Serum Half-Life of G-CSF via Fusion with the Domain III of Human Serum Albumin

**DOI:** 10.1155/2013/107238

**Published:** 2013-09-15

**Authors:** Shuqiang Zhao, Yu Zhang, Hong Tian, Xiaofei Chen, Di Cai, Wenbing Yao, Xiangdong Gao

**Affiliations:** State Key Laboratory of Natural Medicines, School of Life Science and Technology, China Pharmaceutical University, Tongjiaxiang 24, Nanjing 210009, China

## Abstract

Protein fusion technology is one of the most commonly used methods to extend the half-life of therapeutic proteins. In this study, in order to prolong the half-life of Granulocyte colony stimulating factor (G-CSF), the domain III of human serum albumin (3DHSA) was genetically fused to the N-terminal of G-CSF. The 3DHSA-G-CSF fusion gene was cloned into *p*PICZ**α**A along with the open reading frame of the **α**-factor signal under the control of the AOX1 promoter. The recombinant expression vector was transformed into *Pichia pastoris GS*115, and the recombinant strains were screened by SDS-PAGE. As expected, the 3DHSA-G-CSF showed high binding affinity with HSA antibody and G-CSF antibody, and the natural N-terminal of 3DHSA was detected by N-terminal sequencing. The bioactivity and pharmacokinetic studies of 3DHSA-G-CSF were respectively determined using neutropenia model mice and human G-CSF ELISA kit. The results demonstrated that 3DHSA-G-CSF has the ability to increase the peripheral white blood cell (WBC) counts of neutropenia model mice, and the half-life of 3DHSA-G-CSF is longer than that of native G-CSF. In conclusion, 3DHSA can be used to extend the half-life of G-CSF.

## 1. Introduction

Human Granulocyte colony stimulating factor (G-CSF) gene was first reported in 1986, and G-CSF is a 174 amino acid glycoprotein belonging to the family of hematopoietic cytokine [1]. G-CSF was first purified from *CHU-2* cells and showed specific activity on stimulation of mainly granulocyte colony formation [2]. 

 In clinical practice, recombinant human G-CSF has been widely used for neutropenia caused by chemotherapy and radiotherapy [3]. Recently, G-CSF has been demonstrated taking profound effect on nervous system diseases, such as Parkinson's disease and Alzheimer's disease [4–6]. Because of the receptor-mediated elimination, kidney clearance, and enzymatic degradation mechanism, the half-life of G-CSF is so short that patients had to object frequently intravenous infusions or high dose administration of G-CSF to maintain the effective concentration [7, 8]. Therefore, it is very necessary to develop an effective strategy to generate long acting recombinant G-CSF at low price. 

Several approaches have been used to extend the half-life of drugs, and protein fusion technology is one of the most commonly used methods to prolong the half-life of protein and peptide drugs. Based on the development of molecular biology and genetic engineering, some natural proteins with long half-life have been used as fusion partners to enhance the circulating half-life of drugs, such as IgG-Fc, transferrin, and human serum albumin (HSA) [9–11]. There are many successful studies on therapeutic drugs of clinical interest which were fused to HSA and expressed in *P. pastoris *[12]. Many efforts have been done to investigate the mechanism of the long circulating time of HSA in serum. Eventually, researchers found that the retention time of HSA (up to 19 days) mainly depends on the tight interaction between HSA and the neonatal Fc receptor (FcRn) [13]. In general, FcRn can capture HSA in a pH-dependent manner, which protects HSA from normal lysosomal degradation after being taken into cells [14]. Further investigation indicated that the domain III of HSA (3DHSA) alone is both necessary and sufficient for binding to FcRn, and the histidine residues in 3DHSA could dominate the interaction between HSA and FcRn [15]. So 3DHSA is a potential fusion partner for short-acting drugs. Kenanova et al. had fused 3DHSA to a diabody via an 18 amino acid linker and achieved extended serum persistence [16]. However, the related research has not been carried out widely. So, further study is urgent to inspect the effect of 3DHSA for another effective molecule which is limited by short half-life.

In this study, in order to prolong the half-life of G-CSF, we genetically fused it with 3DHSA and then successfully expressed the 3DHSA-G-CSF fusion protein in *P. pastoris GS*115. To our knowledge, this is the first report of 3DHSA-G-CSF expression in any expression system. The biological activity and pharmacokinetic property of the purified fusion protein were also studied utilizing neutropenia model mice and normal mice, and the results indicated that 3DHSA-G-CSF may be considered as a drug candidate of neutropenia for further research and development.

## 2. Materials and Methods

### 2.1. Strains, Plasmid, and Animals

*E*. *coli* DH5*α*,* P. pastoris GS*115, and *p*PICZ*α*A vector were purchased from Invitrogen. Both HSA and G-CSF genes were synthesized by Invitrogen (Shanghai, China). Male 7~8-week-old BALB/c and ICR mice were purchased from the Comparative Medical Center of Yangzhou University (Jiangsu, China). The animals used in this study were under a 12 h light-dark cycle, and the environmental temperature was maintained at 25°C. Animal experiments in this study were performed in compliance with the Guidelines for Animal Experimentation issued by the Chinese Association for Laboratory Animals Science and the Standards Relating to the Care and Management of Experimental Animals.

### 2.2. Construction of the Recombinant Expression Vector

HSA gene was used as template for cloning 3DHSA-G10 with the following primers: (P1) 5′-TCT***CTCGAG*** AAGAGAGTGGAAGAGCCTCAGAATTT-AAT-3′ (band and italic corresponding to *Xho*I restriction enzyme site, characters underlined corresponding to Kex2 enzyme site) and (P2) 5′-CCAGCGGGGTCAAACCCAAAGCAGCTTGAG-3′. G-CSF gene was used as template for cloning H10-G-CSF with (P3) 5′-TTTGGGTTTGACCCCGCTGGGACCGGCAAG-3′ and (P4) 5′-CGT***CTCGAG***TTAGGGCTGGGCAAGGTGGC-3′. The C-terminal of the 3DHSA-G10 sequence was genetically fused with the H10-G-CSF to construct 3DHSA-G-CSF fusion gene by overlap PCR with (P1) and (P4). The fusion gene was inserted into the *Xho*I site of *p*PICZ*α*A along with the open reading frame of the *α*-factor signal under the control of the AOX1 promoter. The KEX2 cleavage site, located between the signal peptide and the 3DHSA-G-CSF sequences, enables the release of mature 3DHSA-G-CSF with a predicted molecular weight of 42 kD. The constructed recombinant plasmid named *p*PIC-3DHSA-G-CSF was confirmed by PCR, single restriction, and nucleotide sequencing.

### 2.3. Transformation and Screening of Recombinant Strains

The positive recombinant expression vector was linearized with *Sal* I and transformed into competent* P. pastoris* host strain *GS*115 by electroporation as Wu and Letchworth reported [17]. The bacterial colonies that grown on the MD medium agar plate were cultured and induced as described in *Pichia *Expression Kit (Invitrogen for user manual). At first, protein samples were checked by 12% SDS-PAGE to screen the recombinant strains, and then the sample of highest expression was transferred to nitrocellulose (NC) filter membranes. The Western blotting was carried out with mouse monoclonal antibody, respectively, against human G-CSF and HSA as primary antibodies and horseradish peroxidase-conjugated goat anti-mouse Ig antibody as secondary antibody for detection of the expressed fusion protein.

### 2.4. Protein Expression and Purification

The recombinant strain was cultured in BMGY medium, and methanol was added every 24 h at the final concentration of 1% (v/v) to induce 3DHSA-G-CSF secreted into BMMY medium at 30°C. 3DHSA-G-CSF was purified by Cibacron Blue F3G-A Sepharose and Butyl-Sepharose 4B [18], and the production of purification was detected by SDS-PAGE.

### 2.5. Peptide Mass Fingerprinting

3DHSA-G-CSF was loaded on SDS-PAGE and stained with Coomassie Blue R250. Then the target bands were excised from the gels and digested by Trypsin (20 mg/mL in 50 mM NH_4_HCO_3_). A MALDI TOF MS spectrometer was used to record and identify the intense peaks. Submit the peptide peak lists to Mascot.

### 2.6. N-Terminal Sequence Analysis

After SDS-PAGE, the fusion proteins in the gel were transferred onto polyvinylidene fluoride (PVDF) membrane (Millipore) and subsequently stained with ponceau stain solution. Compared with the prestained protein marker, the corresponding PVDF bands were cut and sequenced using Edman degradation method to analyze the first 10 amino acids of N-terminal.

### 2.7. Circular Dichroism Analysis

The CD spectra of 3DHSA-G-CSF with the concentration of 0.2 mg/mL in 5 mM sodium acetate buffer (pH 5.0) were detected by a Jasco-720 spectropolarimeter over a range of wavelength of 190–250 nm using a 0.2 cm cell.

### 2.8. Bioactivity Assay

As G-CSF is a hematopoietic cytokine that acts on neutrophil lineage cells and activates mature neutrophils, recombinant G-CSF has been widely used for adjuvant chemotherapy. The biological activity of 3DHSA-G-CSF can be evaluated by determining G-CSF activity of increasing WBC counts of neutropenia murine which was induced by subjecting with cyclophosphamide (CTX) which is a commonly used drug for chemotherapy. Thirty BALB/c mice were intraperitoneally injected with CTX at a single dose of 200 mg/kg to induce neutropenia [19]. The mice whose peripheral white blood cells under the 30% of normal level were randomized divided into three groups, negative control group subjected with saline, positive group with 0.25 mg/kg G-CSF, and test group administered with 0.58 mg/kg 3DHSA-G-CSF (as G-CSF equivalent of 0.25 mg/kg). Six mice of per group were subjected with saline, G-CSF, and 3DHSA-G-CSF, and twenty-four hours after administration, WBC counts were determined with Sysmex NE-8000.

### 2.9. Pharmacokinetic Studies

A preliminary pharmacokinetic study was performed using male ICR mice (7~8 weeks old). Sixty mice were randomly divided into two groups, which were, respectively, injected subcutaneously (S.C) with 1 mg/kg of G-CSF and 2.33 mg/kg of 3DHSA-G-CSF (as G-CSF equivalent of 1 mg/kg). For G-CSF group, blood was collected at 0.25, 0.5, 1, 2, 4, 8, 12, 16, and 24 h after injection, and, for 3DHSA-G-CSF group, blood was collected at 0.25, 0.5, 1, 2, 4, 8, 12, 24, and 36 h after injection. Three mice of the two groups were sacrificed at each time point. Blood samples were centrifuged at 3000 rpm for 15 min to obtain serum and then stored at −80°C until analysis. The serum concentrations of G-CSF and 3DHSA-G-CSF were determined by human G-CSF ELISA kit (DKW Co, Shenzhen, China). Plasma concentration data was analyzed with PKSolver [20].

## 3. Results 

### 3.1. Construction of the Recombinant Expression Vector

**Figure 1:**
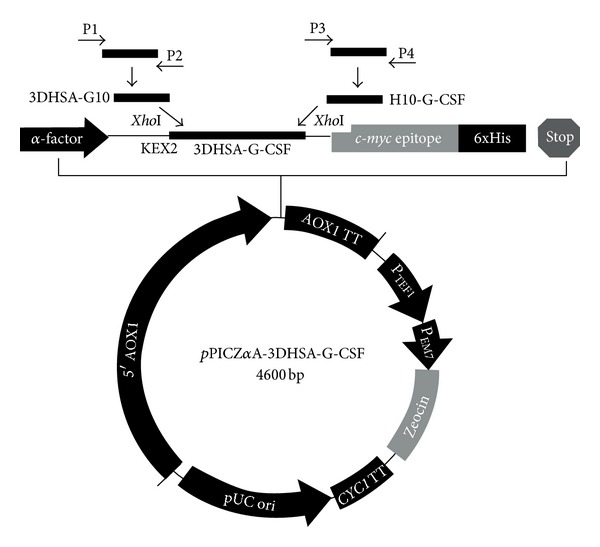
Construction of recombinant *p*PIC-3DHSA-G-CSF eukaryotic expression vector. The synthesized HSA and G-CSF genes were used as templates for the amplification of 3DHSA-G10 and H10-G-CSF genes by PCR with primer 1 and primer 2, primer 3 and primer 4, respectively. The purified 3DHSA-G10 and H10-G-CSF genes were used as templates for obtaining 3DHSA-G-CSF gene by overlap PCR. Both *p*PICZ*α*A plasmid and 3DHSA-G-CSF gene were digested by *Xho*I and ligated together by T4 DNA ligase to construct expression vector *p*PIC-3DHSA-G-CSF. 3DHSA-G-CSF was inserted into the sites *XhoI* of *p*PICZ*α*A along with the open reading frame of the *α*-factor signal under the control of the AOX1 promoter. The KEX2 cleavage site was located between the signal peptide and the 3DHSA-G-CSF sequences.

The 547 bp and 647 bp fragments corresponding to 3DHSA-G10 and H10-G-CSF were amplified by RT-PCR as described above. The full length 3DHSA-G-CSF gene of 1164 bp in size was obtained through overlap PCR, and the purified fusion protein gene was inserted into the expression vector *p*PICZ*α*A to generate a recombinant plasmid *p*PIC-3DHSA-G-CSF. Details of the plasmid *p*PIC-3DHSA-G-CSF and the cloning sites are schematically shown in Figure 1. The recombinant expression vector was confirmed by RT-PCR, single restriction, and nucleotide sequencing (data not shown).

### 3.2. Protein Expression and Purification

**Figure 2:**
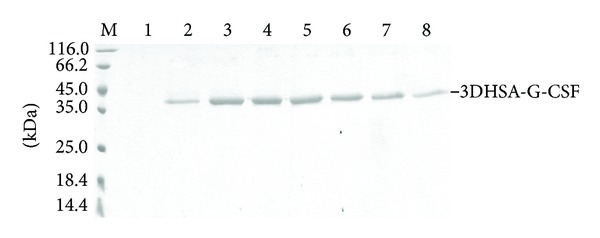
Analysis of transformants that expressed 3DHSA-G-CSF by SDS-PAGE. The culture supernatants of screened transformant were resolved by 12% SDS-PAGE and stained with Coomassie Blue. M: protein marker; Lanes 1–8 correspond to different transformants of *GS*115-*p*PIC-3DHSA-G-CSF; the protein band about 42 kDa was marked 3DHSA-G-CSF on the right of SDS-PAGE.

**Figure 3:**
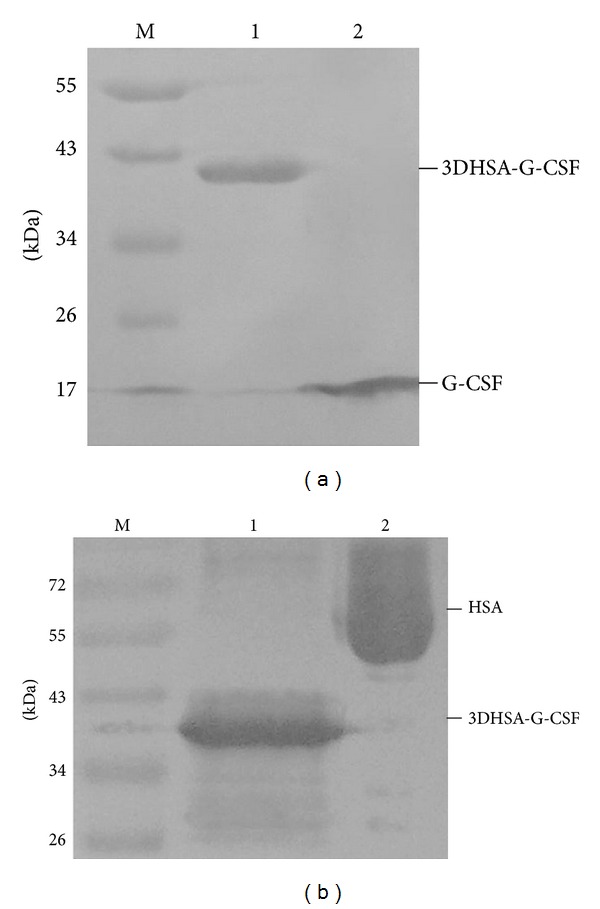
Western blotting analysis using primary antibody anti-G-CSF and anti-HSA. M: prestained protein marker, (a) antibody anti-G-CSF, Lane a1: 3DHSA-G-CSF, Lane a2: G-CSF; (b) antibody anti-HSA, Lane b1: 3DHSA-G-CSF, Lane b2: HSA.

The positive strains expressing fusion protein 3DHSA-G-CSF were screened by 12% SDS-PAGE. As shown in Figure 2, the protein bands of approximately 42 kDa in the gel numbered 2, 3, 4, 5, 6, 7, and 8 are consistent with the theoretical molecular mass of 3DHSA-G-CSF, which indicated that the fusion protein was secreted into supernatant as designed. Western blotting analysis revealed that the fusion protein could be specially recognized by human G-CSF antibody and HSA antibody (Figure 3), suggesting that the protein fusion does not significantly affect the main antigen epitopes.

**Figure 4:**
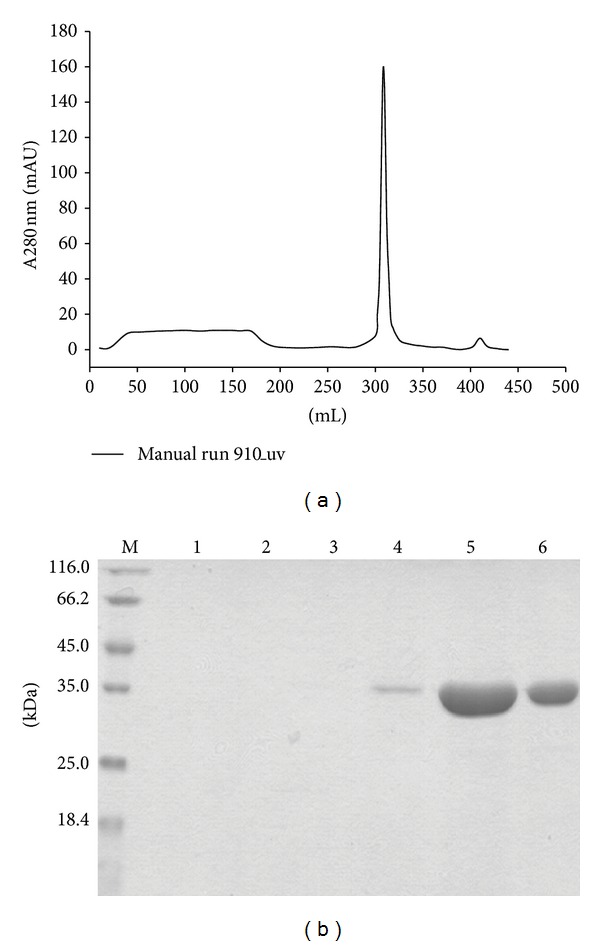
Purification of 3DHSA-G-CSF. The supernatants containing the target proteins were purified by Cibacron Blue F3G-A Sepharose and Butyl-Sepharose 4B column, and the final purity was detected by 12% SDS-PAGE. (a) The hydrophobic chromatograph of 3DHSA-G-CSF by Butyl-Sepharose 4B column. (b) 12% SDS-PAGE, M: protein marker, Lanes 1~3: flow through, Lanes 4~6: elute.

After optimization of the culture conditions, the secretion level of 3DHSA-G-CSF in the broth was 199.5 mg/L. 3DHSA-G-CSF was purified by the combination of affinity chromatography (Cibacron Blue Sepharose Fast Flow column) and hydrophobic chromatography (Butyl Sepharose 4B Fast Flow column), and the products were analyzed by SDS-PAGE. A clear single target band for the fusion protein was shown in Figure 4(b), and the result of Gel scan indicated that the purity of the fusion protein is over 90%. In addition, the final yield of 3DHSA-G-CSF was tested by BCA quantitative protein concentration, and the yield from 1 L medium was 66.2 mg.

### 3.3. Structural Analysis of 3DHSA-G-CSF

**Figure 5:**
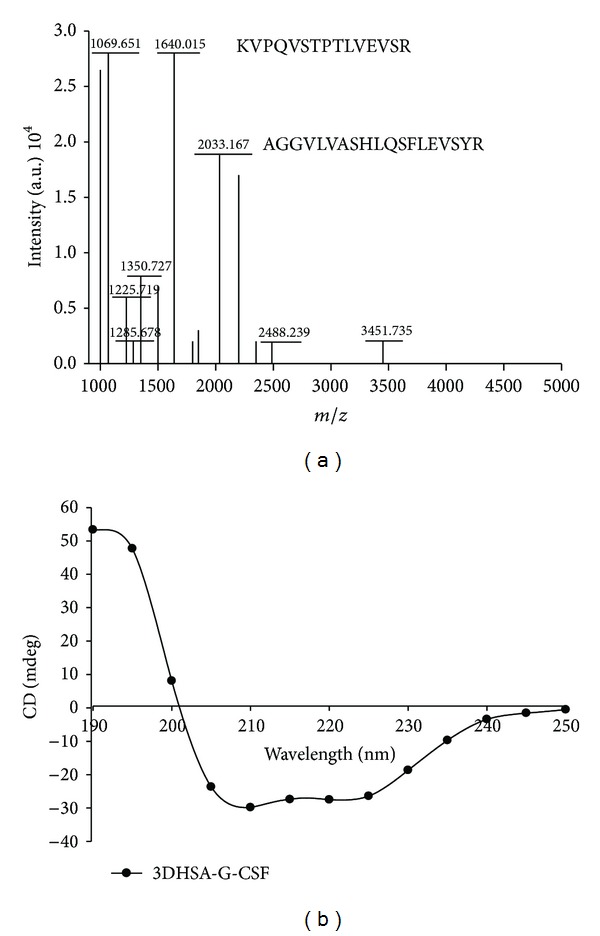
Structural analysis of purified fusion protein by peptide mass mapping and circular dichroism spectrum. (a) Peptide mass mapping of 3DHSA-G-CSF, (b) circular dichroism spectra of 3DHSA-G-CSF.

The peptide mass mapping was adopted to confirm the primary structure of 3DHSA-G-CSF, and the result showed that the peptide fragments were consistent with the theoretical protein sequence, such as KVPQVSTPTLVEVSR and AGGVLVASHLQSFLEVSYR (Figure 5(a)). Meanwhile, it demonstrated that the result of Western blotting is correct and credible. The result of N-terminal sequencing demonstrated that 3DHSA-G-CSF began with the amino acid sequence VEEPQNLIKQ, which represents 100% identity with the theoretical sequence. 

The secondary structure of 3DHSA-G-CSF was evaluated by circular dichroism, and the waveform of 3DHSA-G-CSF was shown in Figure 5(b). The profile of 3DHSA-G-CSF showed the similar shape as HSA-G-CSF, which indicated that 3DHSA-G-CSF was expressed successfully.

### 3.4. *In Vivo* Activity of 3DHSA-G-CSF

**Figure 6:**
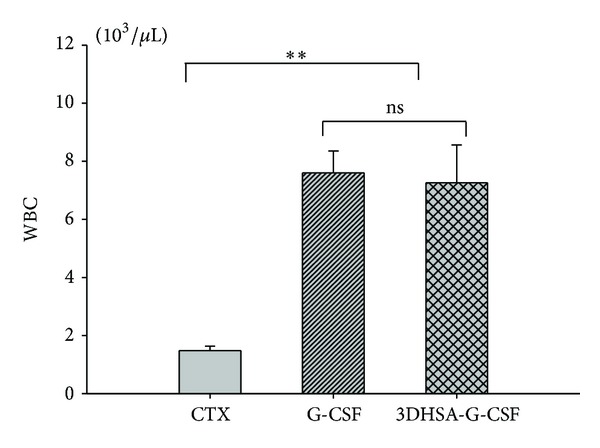
*In vivo* bioactivity of the purified 3DHSA-G-CSF. Both G-CSF and 3DHSA-G-CSF could increase the white blood cell counts in a cyclophosphamide-induced neutropenia model murine, and more potent stimulate was observed compared with CTX (*P* < 0.01). G-CSF versus 3DHSA-G-CSF not significant (ns) (*P* > 0.05). Values are expressed as mean ± SD among six samples from one experiment. Data are representative of three independent experiments with similar result.

Neutropenia model mice were injected with native G-CSF and 3DHSA-G-CSF as described in Section 2. Peripheral white blood cell counts were determined after 24 hours of cytokine injection. As shown in Figure 6, the peripheral WBC counts of both 3DHSA-G-CSF and G-CSF groups were significantly higher than CTX group (*P* < 0.01).

### 3.5. Pharmacokinetic Analysis

**Figure 7:**
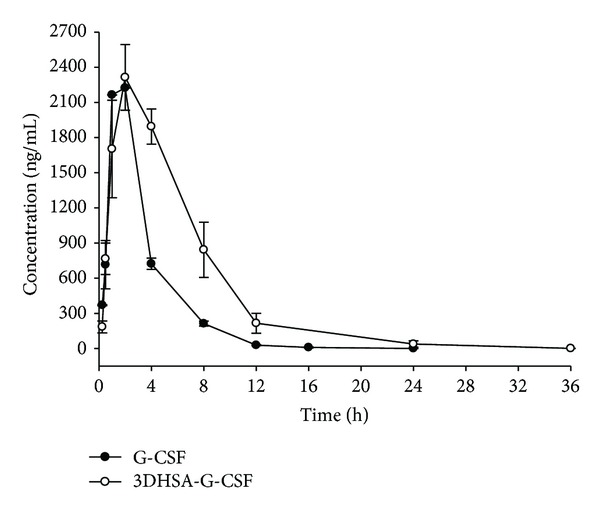
G-CSF level in serum after subcutaneous (S.C.) administration of G-CSF and 3DHSA-G-CSF. The concentration-time curve of 3DHSA-G-CSF is superior than that of G-CSF. Solid black circle means G-CSF. Black hollow circle means 3DHSA-G-CSF. Each point represents the mean value, and error bars represent SD of the mean (*n* = 3). Data are representative of three independent experiments with similar result.

Plasma concentration data of G-CSF and 3DHSA-G-CSF were shown in Figure 7, and the corresponding pharmacokinetic parameters were listed in Table 1. As shown in Figure 7, the concentration-time curve of 3DHSA-G-CSF is superior than that of G-CSF. Furthermore, as shown in Table 1, most pharmacokinetic parameters of 3DHSA-G-CSF are better than G-CSF. Specially, the half-life of G-CSF is only 2.071 ± 0.037 h, and the 3DHSA-G-CSF was determined to be 3.425 ± 0.098 h. Meanwhile, the difference between half-lives of G-CSF and 3DHSA-G-CSF was significant (*P* < 0.01). The data indicated that 3DHSA could be used to extend the half-life of G-CSF.

## 4. Discussions

In the present study, protein fusion technology was adopted to prolong the half-life of G-CSF. 3DHSA was fused with G-CSF to construct recombinant *p*PIC-3DHSA-G-CSF expression vector, and the 3DHSA-G-CSF fusion protein was expressed in *P*. *pastoris* system. *P*. *pastoris* has been developed as an attractive expression platform for heterologous protein production as it grows rapidly and has the ability to accomplish some complex posttranslational modification, such as protein glycosylation, processing, and correct folding. What is more, the very low amount of endogenous proteins secreted by *P. pastoris* represents one of the major advantages of this expression system and serves as the first purification step [21]. Compared to expression G-CSF by methylotrophic yeast *P. pastoris* and G-CSF/IgG-Fc fusion protein in COS-1 cells, 3DHSA-G-CSF was successfully secreted into the supernatant and effectively avoided soluble aggregation product during the fermentation process [9, 22]. In this study, we unexpectedly found that 3DHSA-G-CSF efficiently avoided conspicuous degradation which was common for full-length HSA and albumin fusion protein during the fermentation process [23, 24]. Moreover, other than HSA-G-CSF, no aggregation product was formed during the purification and storage process of 3DHSA-G-CSF [25]. This may be explained by the fact that 3DHSA as a fusion partner has the ability to stabilize the effect molecule [26]. The yield of 3DHSA-G-CSF was much higher than G-CSF and Nartograstim (a derivative of G-CSF) expressed in *E. coli* [27, 28]. This may be also due to 3DHSA, and the result was well supported by the report that fusion partner has the ability to increase the expression level of heterologous protein [29]. 

In order to confirm 3DHSA-G-CSF fusion protein keeps the bioactivity of G-CSF, we determined the *in vivo* activity of 3DHSA-G-CSF using the neutropenia model mice. We observed that both G-CSF and 3DHSA-G-CSF could significantly increase the WBC counts in neutropenia murine model. The result of 3DHSA-G-CSF demonstrated that 3DHSA as a fusion partner did not change the bioactivity of G-CSF and also gave an important evidence for characterization of correct structure of 3DHSA-G-CSF. We next determined the preliminary pharmacokinetic of 3DHSA-G-CSF and G-CSF using normal mice. The half-life of G-CSF is about 2.1 h, while the half-life of 3DHSA-G-CSF is about 3.4 h. We can find that the half-life of G-CSF was prolonged by fusing with 3DHSA. The result also well agrees with the report that 3DHSA is necessary and sufficient for the long serum persistence of albumin [16, 30]. We considered that 3DHSA-G-CSF keeps the ability to bind to FcRn just as G-CSF/IgG-Fc fusion protein. Although the half-life of G-CSF/IgG-Fc is longer than 3DHSA-G-CSF, the dimerization of G-CSF/IgG-Fc fusion proteins is a potential safety concern due to the increased immunogenicity [31]. From the present work it is expected that further preclinical and animal experiments are necessary to prove the efficiency and safety of 3DHSA-G-CSF. In addition, a proof of dynamic bind to their receptors and detailed pharmacokinetic characterizations compared with PEG-G-CSF would further advance the alternatively clinical application of this compound in neutropenia. 

In conclusion, we successfully expressed 3DHSA-G-CSF fusion protein for the first time. The strategy established above suggested that *P*. *pastoris* is an efficient host for 3DHSA-G-CSF expression. 3DHSA-G-CSF is uniform chemical entity which comprising 3DHSA fused to G-CSF, so it may be simpler to manufacture and applied for both research and industrial purpose. Further studies showed that the 3DHSA-G-CSF retained the bioactivity of G-CSF, and its half-life was longer than G-CSF. These data indicated that 3DHSA has the promise to extend the half-life of G-CSF. More importantly, this study provides an experimental evidence for 3DHSA to be applied as a fusion partner to extend the half-life of some other recombinant peptides and proteins in further research.

## Figures and Tables

**Table 1:** Pharmacokinetic parameters of G-CSF and 3DHSA-G-CSF from noncompartmental analysis.

Parameter	Unit	G-CSF	3DHSA-G-CSF
*t*_1/2_	h	2.071 ± 0.037	3.425 ± 0.098**
*T*_max⁡_	h	1.459 ± 0.021	2.097 ± 0.128**
*C*_max⁡_	ng/mL	2224 ± 1.425	2314 ± 280.1
AUC	h·ng/mL	8511 ± 244.7	16339 ± 1316**
MRT	h	3.091 ± 0.113	5.550 ± 0.339**
Vz/F	mL/kg	351.1 ± 5.507	303.3 ± 17.00**
Cl/F	mL/h/kg	117.5 ± 3.414	61.48 ± 5.191**

Values are expressed as mean ± SD (*n* = 3).

SC: subcutaneous; *t*_1/2_: terminal half-life; *T*_max⁡_: time of maximum concentration; *C*_max⁡_: maximum concentration; AUC: area under the curve; MRT: mean residence time; Vz/F: volume of distribution over bioavailability; CL/F: clearance over bioavailability.

The half-life of 3DHSA-G-CSF is longer than G-CSF and showed significant.

**P < 0.01 versus G-CSF.

## Abbreviations

G-CSF: Granulocyte colony stimulating factor
HSA: Human serum albumin
3DHSA: Domain III of human serum albumin
WBC: White blood cell
S.C.: Subcutaneously
*t*_1/2_: Terminal half-life
*T*_max⁡_: Time of maximum concentration
*C*_max⁡_: Maximum concentration
AUC: Area under the curve
MRT: Mean residence time
Vz/F: Volume of distribution over bioavailability
CL/F: Clearance over bioavailability.
